# Does preoperative opioid use predict outcomes to 6 months following primary unilateral knee or hip arthroplasty for osteoarthritis? A data-linked retrospective study

**DOI:** 10.1186/s42836-024-00234-6

**Published:** 2024-03-05

**Authors:** Furkan Genel, Ian A. Harris, Natalie Pavlovic, Adriane Lewin, Rajat Mittal, Andrew Y. Huang, Jonathan Penm, Asad E. Patanwala, Bernadette Brady, Sam Adie, Justine M. Naylor

**Affiliations:** 1 Whitlam Orthopaedic Research Centre, Ingham Institute for Applied Medical Research, Sydney, NSW 2170 Australia; 2 St. George and Sutherland Clinical School, Faculty of Medicine and Health, University of New South Wales, Sydney, NSW 2217 Australia; 3 Liverpool Hospital, South Western Sydney Local Health District, Sydney, NSW 2170 Australia; 4 South Western Sydney Clinical School, Faculty of Medicine and Health, University of New South Wales, Sydney, NSW 2170 Australia; 5 Fairfield Hospital, South Western Sydney Local Health District, Sydney, NSW 2176 Australia; 6 Melbourne Medical School, Faculty of Medicine, Dentistry and Health Sciences, The University of Melbourne, Parkville, VIC 3010 Australia; 7 School of Pharmacy, Faculty of Medicine and Health, The University of Sydney, Sydney, NSW 2050 Australia; 8 Department of Pharmacy, Prince of Wales Hospital and Community Health Services, Randwick, NSW 2031 Australia; 9 Department of Pharmacy, Royal Prince Alfred Hospital, Camperdown, NSW 2050 Australia; 10 Faculty of Medicine and Health, The University of Sydney, Sydney, NSW 2050 Australia; 11 School of Health Sciences, Western Sydney University, Sydney, NSW 2751 Australia; 12 St. George and Sutherland Centre for Clinical Orthopaedic Research, Kogarah, NSW 2217 Australia

**Keywords:** Opioids, Total knee arthroplasty, Total hip arthroplasty, Postoperative outcomes, Retrospective study

## Abstract

**Background:**

Few Australian studies have examined the incidence of prescribed opioid use prior to primary total knee or total hip arthroplasty (TKA, THA) and whether it predicts post-surgery outcomes. A recent Australian study demonstrated that the prevalence of pre-arthroplasty opioid use was approximately 16%. In the United States, approximately 24% of people undergoing TKA or THA are chronic opioid users preoperatively.

**Purpose:**

This study aimed to determine (i) the proportion of TKA and THA patients who use prescribed opioids regularly (daily) before surgery (i.e., opioid use reported between the time of waitlisting and any time up to 3 months before surgery), (ii) if opioid use before surgery predicts (a) complication/readmission rates to 6-months post-surgery, and (b) patient-reported outcomes to 6-months post-surgery.

**Methods:**

A retrospective cohort study of patients who underwent TKA or THA between January 2013 and June 2018 from two Australian public hospitals was undertaken utilizing linked individual patient-level data from two prospectively collected independent databases comprising approximately 3,500 and 9,500 people (database contained known opioid usage data within the 5-year time frame). Inclusion criteria included (i) primary diagnosis of osteoarthritis of the index joint, (ii) primary elective THA or TKA, and (iii) age ≥ 18 years. Exclusion criteria included (i) revision arthroplasty, (ii) non-elective arthroplasty, (iii) hip hemiarthroplasty, (iv) uni-compartmental knee arthroplasty, and (v) previous unilateral high tibial osteotomy.

**Results:**

Analysis was completed on 1,187 study participants (64% female, 69% TKA, mean (SD) age 67 [9.9]). 30% were using regular opioids preoperatively. Adjusted regression analyses controlling for multiple co-variates indicated no significant association between preoperative opioid use and complications/readmission rates or patient-reported outcomes to 6 months post-surgery. Model diagnostics produced poor discrimination for area under the curves and non-significant goodness of fit tests. Pre-arthroplasty opioid use was associated with lower health-related quality of life (EuroQol-Visual Analogue Scale) compared to non-opioid users undergoing primary THA (mean difference -5.04 [-9.87, -0.22], *P* = 0.04, Adjusted R^2^ = 0.06)

**Conclusion:**

In this study, 30% of patients were using prescribed opioids daily prior to primary TKA or THA. Pre-arthroplasty opioid use was not associated with postoperative adverse events or patient-reported pain, function, or global perceived improvement up to six months post-surgery.

**Supplementary Information:**

The online version contains supplementary material available at 10.1186/s42836-024-00234-6.

## Introduction

Opioids are often used to manage osteoarthritic pain [1–4]. Over the last two decades, opioid use has gradually increased globally [5], with Australia [1, 6] and other countries [7, 8] considered to be experiencing an opioid crisis. Specifically, hospitalizations and deaths related to opioids have steadily increased since 2000 [9], although signs of improvement have emerged since 2015-16 [10]. A recent meta-analysis estimated that 24% of total knee arthroplasty (TKA) or total hip arthroplasty (THA) patients are using opioids leading up to surgery. Patients on opioids prior to arthroplasty had worse absolute postoperative patient patient-reported outcome scores compared to those who are opioid naive [11].

Chronic use of opioids preoperatively is associated with an increased risk of persistent use post-operatively [12–14] as well as postoperative complications such as increased surgical site infections and revision arthroplasty [15–19]. Chronic pre-operative opioid users are more likely to require significantly higher opioid doses in the perioperative period due to increased analgesic requirements [20]. This is of concern as increased opioid use in the early post-arthroplasty period (days 1–3 following TKA or THA) is associated with increased venous thromboembolic events and prosthetic joint infections in a dose-dependent fashion [21]. A recent Australian study found a prevalence of approximately 16% of daily prescribed opioid use prior to arthroplasty, and opioid use was associated with increased acute post-arthroplasty complications in unadjusted analysis [22]. Research assessing the risks associated with pre-arthroplasty opioid use and postoperative outcomes (such as complications, readmissions, and patient-reported outcome measures) is mainly available in the non-Australian context [11]. These studies are predominantly from populations in the United States (US), who suffer from higher rates of opioid use compared to Europe and Australasia [23]. Furthermore, the US studies [11] recruited patients between 2000–2014, whilst the Australian study [22] recruited patients between 2018–2019.

Using two independent clinical databases, this retrospective study aimed to assess the relationship between opioid incidence and surgical outcomes in the Australian context. The primary aims were to (i) describe the proportion of patients who were using prescribed opioids daily prior to total knee or hip arthroplasty (TKA or THA) and (ii) determine whether daily preoperative opioid use was associated with complications and readmissions up to 6 months following surgery. The secondary aims were to determine whether pre-arthroplasty opioid use predicted (a) Oxford Hip or Knee Scores (OHS/OKS) [24], (b) Global perceived improvement scale [25], and (c) EuroQol Visual Analogue Scale (EQ-VAS) [26] at 6 months following surgery.

## Materials and methods

Ethics approval was obtained through the South Western Sydney Local Health District (SWSLHD) Human Research Ethics Committee (approval number 2020/ETH01867). The Strengthening the Reporting of Observational Studies in Epidemiology guidelines were used in reporting this study [27]. We retrospectively searched the Arthroplasty Clinical Outcome Registry National (ACORN) [25] and South Western Sydney’s Osteoarthritis Chronic Care Program (OACCP) [28] datasets involving patients undergoing primary THA or TKA between January 2013 and June 2018 at two New South Wales (NSW) public hospitals (Fairfield and Bowral).

**Fig. 1:**
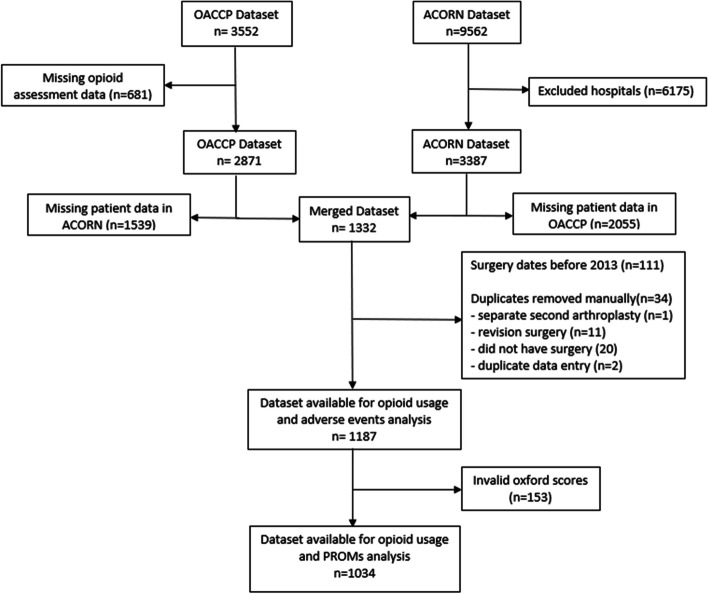
Cohort ascertainment flow diagram

The person-level inclusion criteria included (1) primary diagnosis of osteoarthritis in the index joint, (2) primary elective THA or TKA procedure, (3) age 18 or over, and (4) data available regarding known opioid usage. The exclusion criteria for the index procedure included (1) revision arthroplasty surgery, (2) non-elective arthroplasty, (3) hip hemiarthroplasty, (4) uni-compartmental knee arthroplasty, and (5) previous high tibial osteotomy on the same side. Patient records that contained invalid scores (such as those with erroneous Oxford scores), missing data, had records prior to 2013, or those that were not available in both databases, were excluded from linkage and subsequent analysis (Fig. 1).

### Data from osteoarthritis chronic care program (OACCP)

Both public hospitals were involved in the pre-arthroplasty OACCP implemented for patients awaiting primary THA or TKA. The program involved assessments by clinical staff at the entry to the waitlist for arthroplasty and multiple follow-up appointments (frequency depending on patient comorbidities, falls risk and duration of waiting for surgery). At clinical assessments, multiple data were collected, including known opioid usage, anthropometric measurements (height, weight, body mass index (BMI)), review of medication use, comorbidities and patient-reported outcome measures (PROMs) including OHS/OKS and EQ-VAS. The custodian of the database is SWSLHD.

### Data from arthroplasty clinical outcome registry national (ACORN)

The ACORN registry collected person-level data from eight public and private hospitals across Australia up to 6 months following TKA or THA. The ACORN registry did not collect data regarding known opioid usage amongst participants. OACCP contained preoperative data whilst ACORN contained data pertaining to post-op variables. Variables consistent between both datasets were patient identifiers and comorbidities. ACORN recorded comorbidities as a dichotomous variable at the time of surgery, thus was used in the data analysis. Other outcome variables were not present within both datasets. Variables contained in ACORN include (and are not limited to) age, gender, BMI (calculated from measured height and weight), procedure completed, joint/side replaced, doctor-diagnosed comorbidities (such as heart disease, diabetes, renal failure, depression/anxiety), EQ-VAS, OHS/OKS (presurgery and 6-month post-surgery), American Society of Anaesthesiology (ASA) score, length of stay and adverse events (covering iatrogenic events and post-surgical complications up to 6 months, see Table 2 for example). The Oxford Hip/Knee score questionnaire assessed hip/knee symptom severity in patients undergoing hip/knee arthroplasty. It yields a score between 48 (minimal-no joint symptoms) and 0 (severe symptoms). The EQ-VAS required the respondent to rate their general health from 100 (best health imaginable) to 0 (worst health imaginable) [26]. The global perceived improvement scale required the respondent to rate the change in their joint symptoms from pre-arthroplasty to 6 months post-arthroplasty. Access to the database was obtained through the data custodian, Whitlam Orthopaedic Research Centre (located within Ingham Institute for Applied Medical Research, Liverpool, NSW, Australia).

### Data cleaning and linkage

Data within the two independent databases were cleaned prior to linkage. This included removing duplicate patient entries within each database and identifying data entry errors (refer to R script in Supplementary file). Following cleaning, variables of interest were identified (see below), and identifiers were utilised to merge the ACORN database with the OACCP database. The identifiers utilised in data merging included surname, first name, year of birth, joint and side.

Patient-level data were linked using the R statistical program [29]. Following linkage, exploratory data analysis of the dataset included checking for outliers, modifying variables required for analysis (see below “Variables of Interest”), and assessing the degree of missingness (refer to Supplementary file for R script)

### Variables of interest

The primary exposure variable was known opioid use recorded in the last OACCP assessment before arthroplasty. Opioid use in the dataset was initially recorded as “Yes” or “No” at the baseline assessment. Subsequent OACCP visits recorded usage as either “Started”, “Increased”, ‘Decreased”, or “Ceased”. Based on a combination of these variables, a new variable was created utilising the last recorded known opioid usage before arthroplasty. The dose or type of opioid use was not recorded consistently in the OACCP assessments.

The primary endpoint of interest was a composite outcome combining adverse events and re-admission up to 6 months following surgery. Adverse events/complications were stratified into (i) all adverse events (total adverse events (totalAE)), (ii) all significant adverse events (total significant adverse events (totalSAE)), (iii) acute significant adverse events (acuteSAE), and (iv) late significant adverse events (lateSAE). “Significant” adverse events/complications were determined with consultation with the study team before data analysis (Table 1). TotalSAE was determined by adding acuteSAE and lateSAE. TotalAE was determined by any adverse events/complications or re-admissions up to 6 months following surgery.

**Table 1:** Definitions of acute and post-discharge significant adverse events (coded as acuteSAE and lateSAE)

Acute significant adverse events	Post-discharge significant adverse events
Significant adverse events occurred between surgery and discharge. These included: - Deep Venous Thrombosis - Pulmonary embolism - Fat emboli - Respiratory infection - Cerebrovascular Symptoms - Dislocation - Fracture - Wound dehiscence - Reoperation during index admission - Death - Superficial Surgical site infection (SSI): --> SSI requiring intravenous antibiotic - Deep SSI: --> SSI requiring surgery with no prosthesis removal --> SSI requiring surgery with prosthesis removal	Significant adverse events (from discharge to 6 months post-surgery) that may have needed readmission, surgery, or non-admission. These included: - Deep Venous Thrombosis (index leg, other leg, both legs) - Pulmonary embolism - Manipulation under anaesthetic - Dislocation - Superficial SSI: --> SSI requiring intravenous antibiotics - Deep SSI: --> SSI requiring surgery with no prosthesis removal --> SSI requiring surgery with prosthesis removal - Wound dehiscence - Index joint revision - Joint stiffness - Periprosthetic fracture - Implant fracture - Bleeding - Pain - cardiac complication - Stroke - Respiratory infection

ACORN data collectors were given the option to select all relevant adverse events that occurred for each patient

The secondary endpoints of interest were OHS/OKS (scored 0–48), EQ-VAS (scored 0-100) and global perceived improvement scale (measured on a Likert-scale as “much better; slightly better; same; slightly worse; much worse”) 6 months following surgery. The global perceived improvement scale was collapsed into two categories (“much better” vs. all other responses) as the sample was not adequately powered to conduct ordinal logistic regression analysis [25].

### Sample size

The study analysis was based on a priori sample size analysis using a recent Australian study. This study found the rate of postoperative readmissions or complications to 3 months post-arthroplasty to be 43% amongst opioid users and 38% amongst opioid naïve users [22]. Assuming the opioid naïve cohort to opioid cohort ratio is 5:1, A sample size of 2748 participants would have 80% statistical power to detect a statistically significant (2-tailed, alpha = 0.05) difference in relative risk between the cohorts at 6 months of about 16% (37% vs. 43%). A sample size of 3500 participants (the known sample available in ACORN) would permit analyses adjusting for covariates.

### Statistical analysis

Descriptive statistics (proportions, means, and standard deviations) were utilised to describe the cohort baseline characteristics and the outcome. The Odds Ratio between known preoperative opioid users and opioid naïve patients was calculated for dichotomous outcomes (adverse events and global perceived improvement at 6 months). The mean difference between preoperative opioid users and opioid naïve patients was calculated for continuous outcomes (Oxford scores and EQ-VAS scores at 6-months post-surgery).

Both unadjusted and adjusted analyses were performed for the primary and secondary outcomes. Multivariable linear regression (for continuous outcomes) and multivariable logistic regression (for categorical outcomes) were utilised in the adjusted analyses. Adjusted analyses enabled controlling for variables that were known or suspected confounders. The confounders used in secondary adjusted analyses included age, sex, BMI, preoperative OHS/OKS (as collected in ACORN [25]), comorbidities, ASA score, education level, surgery type/side and other back pain or lower limb joint symptoms affecting mobility. Adverse event adjusted models were controlled for joint, whilst adjusted analyses for PROMs analyses (OKS/OHS and EQ-VAS) had pre-surgery (baseline) OHS/OKS used as a covariate in its analyses. These confounders were included as prior research indicated they may be associated with the outcome of interest and/or post-arthroplasty outcomes [30–37]. Prior research also demonstrated lower patient-reported outcome measures in patients with lower education levels [38]. Post-hoc sensitivity analyses involved analysis by arthroplasty type (TKA vs. THA).

For dichotomous outcomes (totalAE, totalSAE, acuteSAE and lateSAE), model diagnostics were completed by calculating (and plotting) the area under the receiver operating characteristic curve (AUC) [39] as well as conducting the Hosmer and Lemeshow goodness of fit (GOF) test [40, 41]. The AUC values were classified as outstanding discrimination (≥ 0.9), excellent discrimination (0.8–0.89), acceptable discrimination (0.7–0.79), poor discrimination (0.5–0.69) or no discrimination (< 0.5) [41]. The GOF test classified logistic regression models as poor fit (*P* < 0.05) or good fit (*P* ≥ 0.05) [41].

For continuous outcomes (OKS/OHS and EQ-VAS), model diagnostics were completed by calculating adjusted R-squared and inspecting residuals via modelling graphs (i.e. “residuals vs fitted”, “normal Q-Q”, “scale-location”, “residuals vs leverage” (Cook’s distance)). Plotted graphs are found in the Supplementary file. Adjusted R^2^ values were classified according to Cohen’s benchmarks for R^2^ values [42]. Statistical analysis was completed using the R statistical program (R version 4.1.0 (2021-05-18)).

## Results

### Cohort ascertainment and patient characteristics

Figure 1 shows the study sample ascertainment. The OACCP and ACORN datasets had 3552 and 9562 patients, respectively. Following data cleaning and linkage outlined above in Methods as well as applying the inclusion criteria, complete data for 1187 patients were available to assess the association between prescribed opioid usage and postoperative adverse events. After removing invalid Oxford scores from the merged dataset, 1034 patients were available to assess the association between known opioid usage and postoperative PROMs (Fig. 1).

**Fig. 2:**
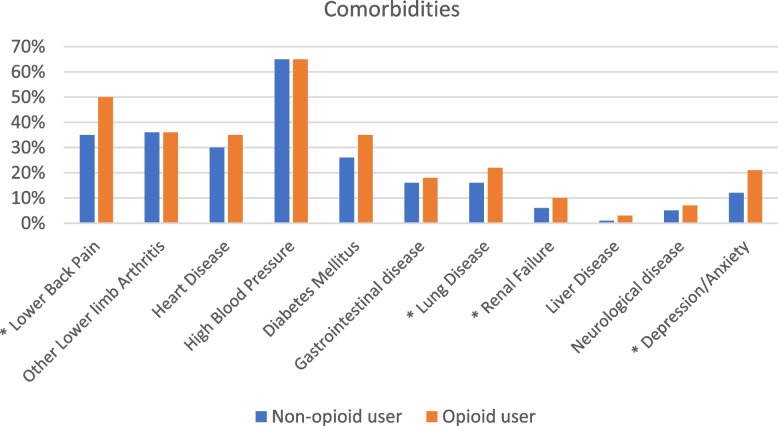
Prevalence of comorbidities amongst non-opioid and opioid users. *: significant difference between the two cohorts,* P* < 0.05

A comparison of baseline characteristics indicated that 30% of patients used prescribed opioids regularly before arthroplasty. The known opioid user cohort (*n* = 359) was found to be younger (mean age 65.2 vs 67.7), have a higher BMI (mean 35.4 vs 32.6), and worse joint symptoms (mean Oxford 12.7 vs 15.9) compared to the non-opioid user cohort (*n* = 824, Table 2). The proportion of patients with ASA 3–4 scores was similar between the two groups (37% vs 36%). There was a lower proportion of patients in the known opioid cohort who had TKA compared to the non-opioid cohort (58% vs 74%, Table 2). Regarding comorbidities, the known opioid user cohort was found to have a higher proportion of patients reporting lower back pain and depression/anxiety (Fig. 2). Other comorbidities were similar between the two cohorts.

**Table 2:** Comparison of the baseline characteristics of the non-opioid and opioid user cohorts

Baseline Characteristic	Non-opioid user (*n* = 824)	Opioid user (*n* = 359)	*P* value
Proportion	70%	30%	-
Female	62%	67%	0.067
Mean age (SD)	67.7 (9.6)	65.2 (10.4)	**< 0.001**
Mean BMI (SD)	32.6 (7.3)	35.4 (23.8)	**0.040**
TKA	74%	58%	**< 0.001**
Right side	50%	49%	0.64
Mean Oxford Score (SD)	15.9 (7.4)	12.7 (6.8)	**< 0.001**
ASA 1–2	64%	62%	0.88
ASA 3–4	36%	37%	0.80

*SD *Standard deviation. Bold values indicate statistical significance (*P* < 0.05)

### Preoperative opioid use as a predictor of postoperative adverse events

Unadjusted and adjusted analyses assessing the association between preoperative opioid use and postoperative adverse events are presented in Tables 3 and 4 respectively. After adjusting for covariates, there was no significant association between preoperative opioid use and the rate of 6-month total and significant adverse events and acute significant adverse events (Table 4). AUC values ranged between 0.63 and 0.66 (poor discrimination). All GOF tests were not statistically significant (i.e. good fit, Table 4).

**Table 3:** Unadjusted analyses assessing the association of pre-operative opioid use and 6-month adverse events and PROMs

**Unadjusted Analysis**	**Non-opioid user** ***n*** **= 824**	**Opioid user** ***n*** **= 359**	***P *** **value**
Total adverse events	38.8%	38.8%	1.00
Acute significant adverse events	5.7%	5.3%	0.88
Post-discharge significant adverse events	6.5%	7.1%	0.78
Total significant adverse events	12.3%	12.7%	0.95
**Unadjusted Analysis**	**Non-opioid user** ***n*** **= 714**	**Opioid user** ***n*** **= 317**	***P*** ** Value**
6-month Oxford Score	39.5 (7.8)	38.8 (8.9)	0.22
6-month EQ-VAS	77.1 (17.8)	71.9 (20.5)	**< 0.001**
6-month “Much Better”	81.1%	80.3%	0.83

Bold values indicate statistical significance (*P* < 0.05)

**Table 4:** Adjusted analyses assessing the association of preoperative opioid use with 6-month adverse events, the proportion of “Much Better” on the global perceived improvement scale, Oxford and EQ-VAS scores

**Adjusted Analysis**	**Opioid user (Odds Ratio (95CI))**	***P *** **value**	**AUC**
Total adverse events	0.85 (0.61, 1.19)	0.35	0.63
Acute significant adverse events	1.05 (0.55, 1.96)	0.87	0.66
Total significant adverse events	0.94 (0.57, 1.50)	0.79	0.64
6-month “Much Better”	0.93 (0.61, 1.43)	0.73	0.64
**Adjusted Analysis**	**Opioid user (MD (95CI))**	***P*** ** value **	**Adjusted R** ^**2**^
6-month Oxford Score	-0.34 (-1.63, 0.95)	0.60	0.08
6-month EQ-VAS	-2.26 (-5.16, 0.61)	0.12	0.09

*95 CI* 95% Confidence intervals. *AUC *Area under the curve, *MD *Mean difference

### Preoperative opioid use as a predictor of postoperative Oxford score, EQ-VAS and global perceived improvement scale

Both unadjusted and adjusted analyses assessing the association between known preoperative opioid use and PROMs resulted in no significant association between opioid use and 6-month Oxford scores (Tables 3 and 4) or the proportion of patients identifying as “Much Better” on the 6-month Global perceived improvement scale (Tables 3 and 4). Model diagnostics for Oxford score produced an adjusted R^2^ value of 0.08 (Table 4, Figure S1), whilst global perceived improvement produced an AUC value of 0.64 (Table 4).

Unadjusted analyses assessing the association between known preoperative opioid use and 6 months EQ-VAS scores resulted in the average opioid user’s EQ-VAS score 5.2 points lower for opioid users than non-opioid users (mean 77.1 vs 71.9, *P* < 0.001, Table 3, minimal clinically important difference for patients undergoing primary TKA is 6.4 [43]). Adjusted analysis controlling for covariates (including pre-surgery EQ-VAS) demonstrated non-significant lower EQ-VAS scores for opioid users (mean difference − 2.26 (-5.16, 0.61), *P* = 0.12, Table 4). Model diagnostics for EQ-VAS produced an adjusted R^2^ value of 0.09 (Figure S2).

### Post-hoc sensitivity analysis by arthroplasty type (TKA vs THA)

Unadjusted and adjusted analyses completed on patients who underwent TKA (Table S1 and S2) or THA (Table S1 and S2) demonstrated no significant association between known preoperative opioid use and 6-month adverse events (totalAE, totalSAE, acuteSAE, lateAE). AUC values ranged between 0.59 and 0.77 (poor-acceptable discrimination). Except for totalAE analysis for TKA, all GOF tests resulted in a *p*-value > 0.05 (Table S1).

Regarding 6-month EQ-VAS scores, unadjusted analyses by arthroplasty type resulted in significantly reduced EQ-VAS scores in known opioid users who underwent TKA (71.3 (20.8) vs 76.3 (18.1), *P* < 0.01, Table S2) and THA (72.7 (20.0) vs 79.3 (16.7), *P* < 0.01, Table S2). Opioid users had significantly lower EQ-VAS in the adjusted THA analysis (-5.04 (-9.87, -0.22), *P* = 0.04, adjusted R^2^ = 0.06, Table 5 and Figure S3).

With regard to Oxford scores, a clinically small but statistically significant difference was found in the TKA unadjusted analysis between non-opioid and known opioid users (38.7 (7.7) vs 37.0 (9.2), *P* = 0.02, Table S2). However this difference was not significant in the adjusted analysis (adjusted R^2^ = 0.025, Table 5, Figure S4).

**Table 5:** Adjusted analyses assessing the association of preoperative opioid use with Oxford and EQ-VAS scores

Arthroplasty Type	Adjusted Analysis	Opioid user (MD (95CI))	*P*-value	Adjusted R^2^
TKA	6-month Oxford Score	-0.46 (-2.03, 1.12)	0.57	0.025
TKA	6-month EQ-VAS	-2.06 (-5.63, 1.51)	0.26	0.058
THA	6-month Oxford Score	-0.45 (-2.63, 1.72)	0.68	0.0066
THA	6-month EQ-VAS	-5.04 (-9.87, -0.22)	**0.04**	0.058

MD (95 CI) = mean difference (95% confidence interval). Bold values indicate statistical significance (*P* < 0.05)

## Discussion

In this retrospective study, 30% of patients were using prescribed opioids daily prior to primary TKA or THA. After adjusting for known confounders, there was no association between daily preoperative opioid use and (i) adverse events up to 6 months following arthroplasty, (ii) Oxford scores, EQ-VAS and global perceived improvement scale at 6 months post arthroplasty. Adjusted post-hoc subgroup analysis found opioid users undergoing THA had reduced 6-month EQ-VAS scores.

The proportion of known opioid users in our study was high, consistent with Australian data indicating increasing opioid use within the study period analysed in our analysis [44]. This high proportion is also consistent with data from the US (24%) [11]. A recent Australian study demonstrated the prevalence of opioid use prior to surgery to be approximately 16% [22]. More recent Australian [22, 23] and US [45] data indicate the rates may have reduced over time. Decreases in rates over time may be explained by the fact that the Australian study had data from 2018–2019, whilst our study had data from 2013–2018. There had been changes in regulations for dispensing opioids and policy changes made by the Royal Australasian College of General Practitioners concerning community opioid prescribing. These changes made from 2015–2018 [46] may explain the reduced levels of opioid use in 2018–2019 in SWSLHD. Another recent Australian prospective study demonstrated a prevalence of 19% of prescribed opioid use prior to hip or knee arthroplasty [23]. However, the patients recruited for that study were all privately insured and had surgeries through private hospitals. Surgeries in the private sector occur sooner than those awaiting surgery through the public health system [23]. Public patients experiencing increased wait times for arthroplasty in the public health system may result in worse symptoms as well as progression of disease complexity [47, 48], which may result in a higher prevalence of opioid use to manage their pain and maintain function. Furthermore, public patients in SWSLHD come from low socioeconomic class [49], thus increasing the prevalence of opioid use prior to arthroplasty [50].

In our study, known pre-arthroplasty opioid use was not associated with postoperative adverse events. This is consistent with a previous Australian study conducted in a public hospital [22]. However, recent systematic reviews concluded that pre-arthroplasty opioid use is associated with an increased risk of readmission, prosthetic joint infection, revision arthroplasty [45], and worse postoperative PROMs at 6 months [11]. Furthermore, recent US studies demonstrated that pre-TKA opioid use increased post-TKA complications in a dose-dependent manner [51, 52]. In this study, we did not have adequate data to assess whether a dose-dependent relationship exists.

Our study did not observe worse patient-reported outcomes overall in prescribed opioid users at 6 months post arthroplasty. Regarding EQ-VAS, our study demonstrated a reduced EQ-VAS score at 6 months post-arthroplasty for opioid users undergoing THA, contrary to previous research which demonstrated no difference in EQ-VAS scores between known opioid users and non-opioid users following arthroplasty [53]. This difference in EQ-VAS scores may be explained by differences in the follow-up time between our study (6 months post arthroplasty) and the previously mentioned study (1-1.5 years post arthroplasty [53]).

Our retrospective study included participants recruited between 2013–2018, data was sourced from two databases and opioid usage was determined as the last recorded known usage of opioids prior to arthroplasty. Goplen’s meta-analysis [11] included studies from 2010–2017, where participants were recruited between 2000–2014. The meta-analysis included 5 retrospective studies that had data collected prospectively and follow up ranged from 6 months to 58 months. Data sources included databases as well as studies conducted at institutions. In these studies, opioid usage was determined as either opioids prescribed within the last 2 years prior to surgery, prescribed opioids at time of surgery (two studies), or known usage of opioids for a given time prior to surgery (one study determined as 4 weeks whilst another as 6 weeks [11]). Moreover, Chen’s meta-analysis [45] included studies published from 2006–2017 where participant data was sourced from either databases or institutions between 2003–2016. Participants follow-up period lasted for 6 months to 3 years [45].

This study was conducted in the Australian context, whilst all of the studies included in the two aforementioned systematic reviews [11, 45] were conducted in the US. This may explain the differences in the postoperative adverse outcomes and patient-reported outcomes published in the literature as THA patients from the US were found to be younger, heavier, more comorbid and less likely to have their surgery at high-volume hospitals (> 300 THAs/year) compared to Australian patients [54] (clinically relevant as superior clinical outcomes have been demonstrated in patients undergoing THA in high volume hospitals/centres [55]).

### Strengths and limitations

Strengths of this study included (i) the patient-level linkage of two separate existing clinical datasets, (ii) the utilization of datasets that were created for the purpose of measuring outcomes of total joint replacement and patient characteristics, and (iii) data within the datasets were recorded prospectively. Regarding limitations, the initial study intention was to assess a dose-effect relationship between opioids and adverse events. However, following a review of the datasets utilized in this study, this was abandoned as the data did not allow for rigorous stratification and subsequent analysis due to data deficiencies. Furthermore, the exposure variable was dichotomous (i.e., opioid use prior to surgery was deemed as “yes” or “no”). We could not differentiate between low and high prescribed opioid users to determine whether there was a dose-dependent association between opioid use and adverse events postoperatively; This did not allow for rigorous stratification and subsequent analysis due to data deficiencies. Finally, data used in this study did not capture persistent opioid use following TKA or THA nor the effects of persistent post-arthroplasty opioid use on adverse events and patient-reported outcome measures. Future research is needed to assess the relationship between the amount/dose of daily pre-arthroplasty opioid use and the risk of adverse events and PROMs.

## Conclusion

In this retrospective study, 30% of patients were using prescribed opioids daily prior to primary TKA or THA. Opioid use prior to surgery was not associated with adverse events or patient-reported pain, function, or improvement up to six months post-surgery. However, known opioid use prior to surgery was associated with lower health-related quality of life (EQ-VAS) compared to non-opioid users undergoing primary THA.

### Supplementary Information

**Additional file 1: Figure S1.** Model Diagnostics for the association between Opioid use and Oxford Score. **Figure S2.** Model Diagnostics for the association between Opioid use and EQ VAS Score. **Figure S3. **Model Diagnostics for the association between Opioid use and EQ VAS – THA Subgroup analysis.**Figure S4.** Model Diagnostics for the association between Opioid use and Oxford Score – TKA subgroup analysis. **Table S1.** Association between pre-operative opioid use and 6-month adverse events by arthroplasty type (TKA or THA). AE = adverse events, OR = Odds Ratio, 95CI = 95% Confidence intervals, AUC = Area under the curve. **Table S2.** Unadjusted analyses assessing the association of pre-operative opioid use and 6-month adverse events in patients who underwent TKA. STROBE Statement—Checklist of items that should be included in reports of case-control studies [[relevant pages checked against the checklist are found within the brackets below next to each item description]].

## Data Availability

Study data and materials are held by the researchers at Whitlam Orthopaedic Research Centre. The datasets used and analyzed during the current study are available from the data custodians upon reasonable request.

## Abbreviations

95CI: 95% Confidence interval
ACORN: Arthroplasty Clinical Outcome Registry National
AcuteSAE: Acute significant adverse events
AUC: Area under the curve
BMI: Body mass index
EQ-VAS: EuroQol Visual Analogue Scale
GOF: Hosmer and Lemeshow goodness of fit test
LateSAE: Late significant adverse events
MD: Mean difference
NSW: New South Wales
OACCP: Osteoarthritis Chronic Care Program
OHS: Oxford Hip Score
OKS: Oxford Knee Score
PROMs: Patient-reported outcome measures
SD: Standard Deviation
SSI: Surgical site infection
SWSLHD: South Western Sydney Local Health District
THA: Total hip arthroplasty
TKA: Total knee arthroplasty
TotalAE: Total adverse events
TotalSAE: Total significant adverse events
US: the United States
