# Supplementary material for: Does preoperative opioid use predict outcomes to 6 months following primary unilateral knee or hip arthroplasty for osteoarthritis? A data-linked retrospective study
Source: Arthroplasty. 2024 Mar 5;6:11. doi: 10.1186/s42836-024-00234-6 (PMC10913630; doi:10.1186/s42836-024-00234-6)
Supplement: Supplementary file 1 — Additional file 1: Figure S1. Model Diagnostics for the association between Opioid use and Oxford Score. Figure S2. Model Diagnostics for the association between Opioid use and EQ VAS Score. Figure S3. Model Diagnostics for the association between Opioid use and EQ VAS – THA Subgroup analysis.Figure S4. Model Diagnostics for the association between Opioid use and Oxford Score – TKA subgroup analysis. Table S1. Association between pre-operative opioid use and 6-month adverse events by arthroplasty type (TKA or THA). AE = adverse events, OR = Odds Ratio, 95CI = 95% Confidence intervals, AUC = Area under the curve. Table S2. Unadjusted analyses assessing the association of pre-operative opioid use and 6-month adverse events in patients who underwent TKA. STROBE Statement—Checklist of items that should be included in reports of case-control studies [[relevant pages checked against the checklist are found within the brackets below next to each item description]]. [file 42836_2024_234_MOESM1_ESM.docx]

Figure S1: Model Diagnostics for the association between Opioid use and Oxford Score
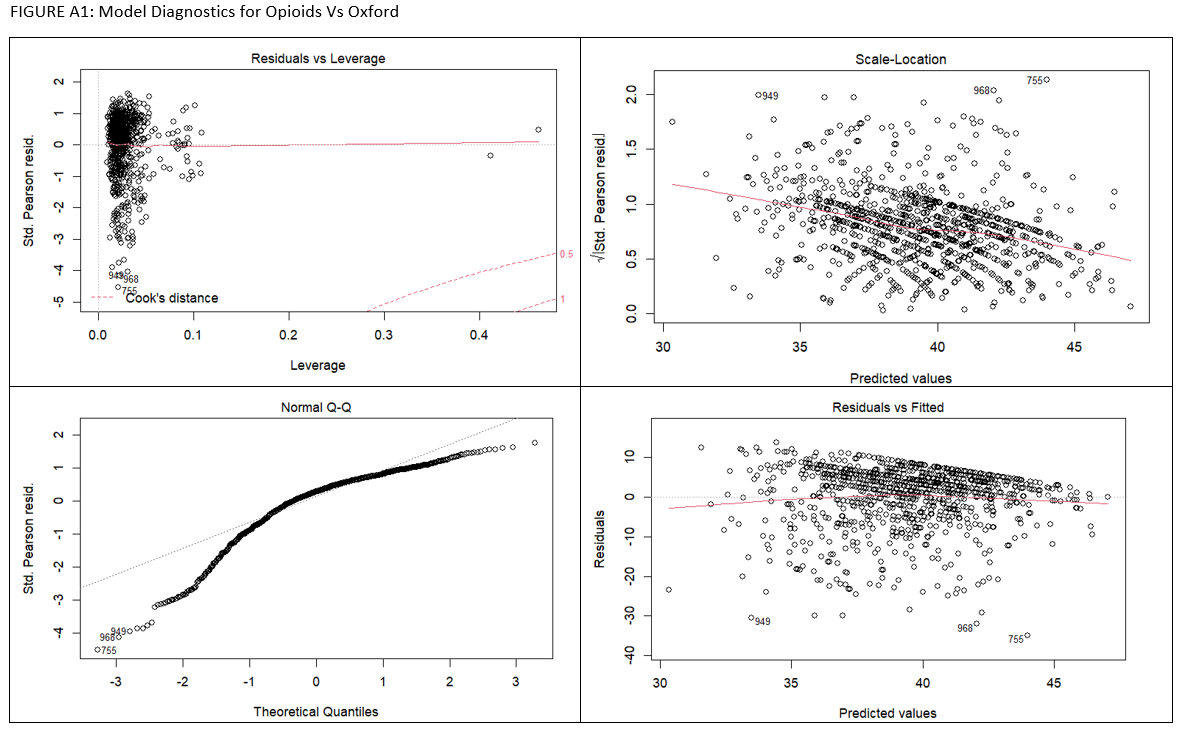


Figure S2: Model Diagnostics for the association between Opioid use and EQ VAS Score


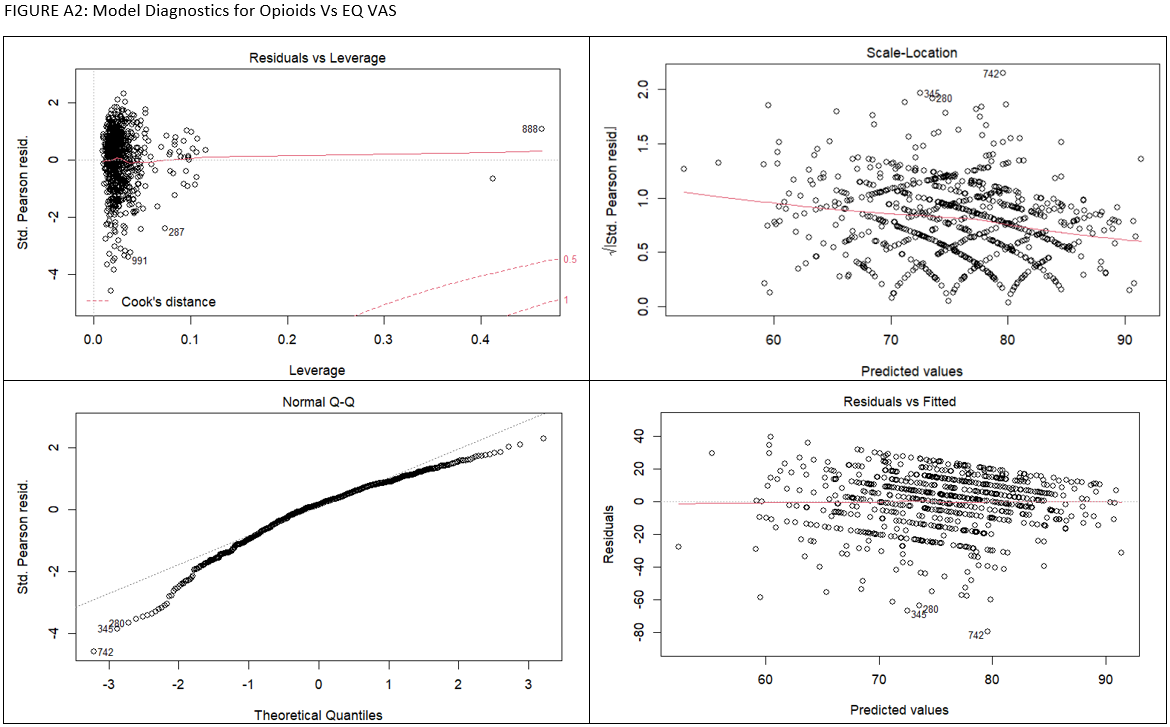


Figure S3: Model Diagnostics for the association between Opioid use and EQ VAS – THA Subgroup analysis


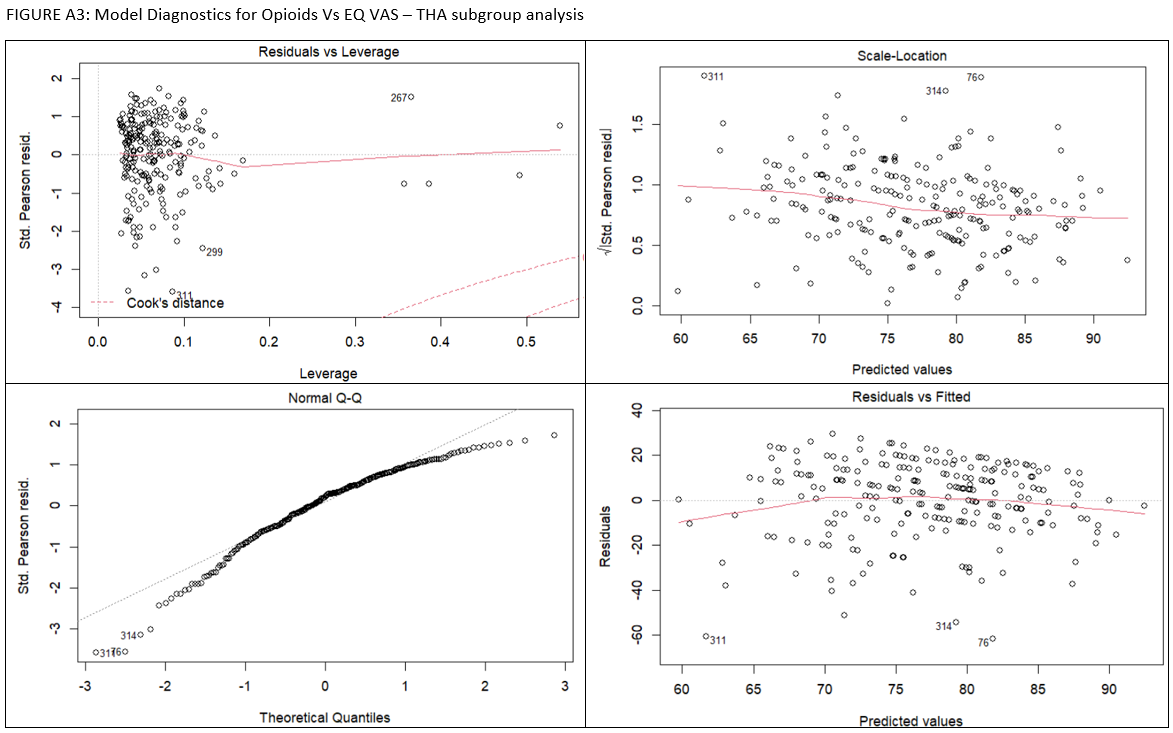


Figure S4: Model Diagnostics for the association between Opioid use and Oxford Score – TKA subgroup analysis


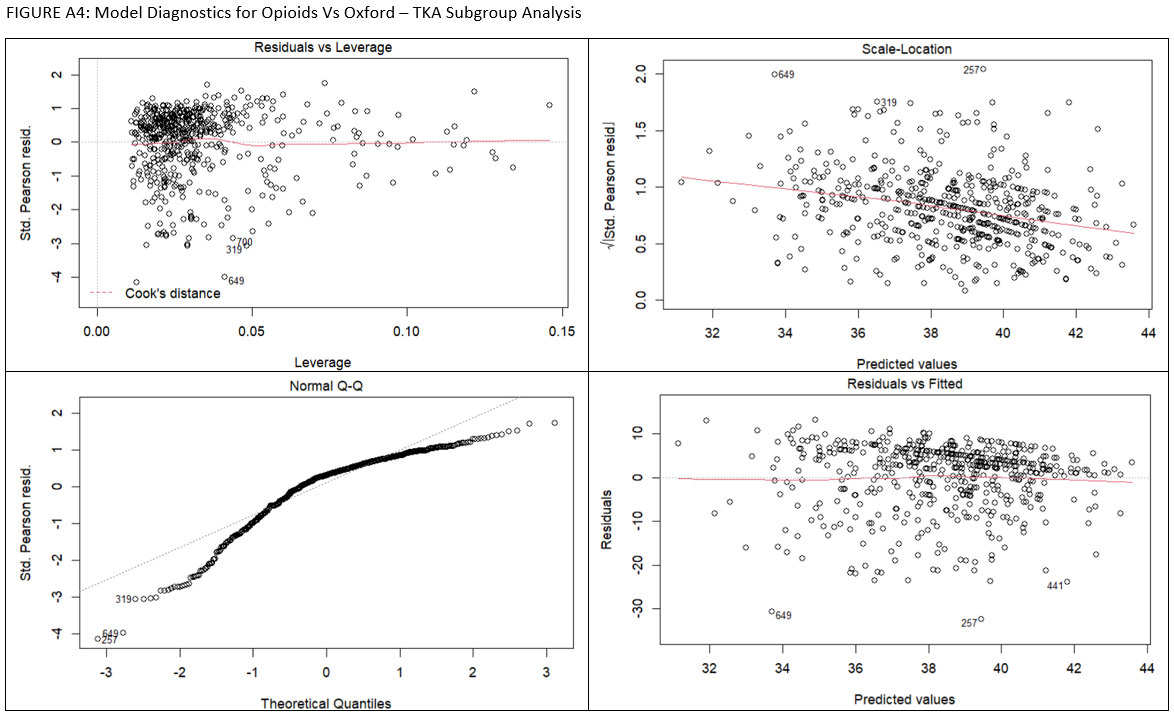


Table S1: Association between pre-operative opioid use and 6-month adverse events by arthroplasty type (TKA or THA).

| **Arthroplasty type** | **Adjusted Analysis** | **Opioid user**  **(OR (95CI))** | **P-value** | **AUC** |
| --- | --- | --- | --- | --- |
| TKA | Total AEs | 0.77 (0.51, 1.17) | 0.23 | AUC = 0.64 |
| TKA | Acute Significant AEs | 0.72 (0.26, 1.74) | 0.50 | AUC = 0.69 |
| TKA | Total significant AEs | 0.85 (0.44, 1.55) | 0.61 | AUC = 0.67 |
| TKA | 6-month “Much Better” | 1.14 (0.71, 1.89) | 0.59 | AUC = 0.59 |
| THA | Total AEs | 0.94 (0.49, 1.77) | 0.85 | AUC = 0.65 |
| THA | Acute Significant AEs | 0.81 (0.20, 2.92) | 0.76 | AUC = 0.77 |
| THA | Total significant AEs | 0.73 (0.26, 1.87) | 0.52 | AUC = 0.71 |
| THA | 6-month “Much Better” | 0.50 (0.21, 1.14) | 0.10 | AUC = 0.69 |

AE = adverse events, OR = Odds Ratio, 95CI = 95% Confidence intervals, AUC = Area under the curve.

Table S2*: Unadjusted analyses assessing the association of pre-operative opioid use and 6-month adverse events in patients who underwent TKA.*

| **Unadjusted Analysis**  **TKA** | **Non-opioid user**  **N=526** | **Opioid user**  **N=187** | **P-value** |
| --- | --- | --- | --- |
| Total AEs | 41.5% | 40.5% | 0.90 |
| Acute Significant AEs | 6.1% | 4.3% | 0.46 |
| Post-discharge Significant AEs | 6.9% | 8.0% | 0.73 |
| Total Significant AEs | 12.6% | 12.3% | 1.00 |
| 6-month Oxford Score | 38.7 (7.7) | 37.0 (9.2) | **0.02** |
| 6-month EQ-VAS | 76.3 (18.1) | 71.3 (20.8) | **<0.01** |
| 6-month “Much Better” | 78.8% | 77.5% | 0.81 |
| **Unadjusted Analysis**  **THA** | **Non-opioid user**  **N=188** | **Opioid user**  **N=130** | **P-value** |
| Total AEs | 31.0% | 32.8% | 0.83 |
| Acute Significant AEs | 5.9% | 4.6% | 0.82 |
| Post-discharge Significant AEs | 6.5% | 6.9% | 1.00 |
| Total Significant AEs | 12.4% | 11.5% | 0.96 |
| 6-month Oxford Score | 41.7 (8.0) | 41.4 (7.4) | 0.70 |
| 6-month EQ-VAS | 79.3 (16.7) | 72.7 (20.0) | **<0.01** |
| 6-month “Much Better” | 87.7% | 84.4% | 0.49 |

STROBE Statement—Checklist of items that should be included in reports of ***case-control studies*** *[[relevant pages checked against the checklist are found within the brackets below next to each item description]]*

|  | **Item No** | | **Recommendation** |
| --- | --- | --- | --- |
| **Title and abstract** | 1 | | (*a*) Indicate the study’s design with a commonly used term in the title or the abstract [[PAGE 1]] |
|  |  |  | (*b*) Provide in the abstract an informative and balanced summary of what was done and what was found [[PAGES 2-3]] |
| **Introduction** | | | |
| Background/rationale | 2 | | Explain the scientific background and rationale for the investigation being reported [[PAGES 4-5]] |
| Objectives | 3 | | State specific objectives, including any prespecified hypotheses [[PAGE 5]] |
| **Methods** | | | |
| Study design | 4 | | Present key elements of study design early in the paper [[PAGE 5]] |
| Setting | 5 | | Describe the setting, locations, and relevant dates, including periods of recruitment, exposure, follow-up, and data collection [[PAGE 5]] |
| Participants | 6 | | (*a*) Give the eligibility criteria, and the sources and methods of case ascertainment and control selection. Give the rationale for the choice of cases and controls [[PAGE 5-6]] |
|  |  |  | (*b*) For matched studies, give matching criteria and the number of controls per case[[NA]] |
| Variables | 7 | | Clearly define all outcomes, exposures, predictors, potential confounders, and effect modifiers. Give diagnostic criteria, if applicable [[PAGE 7-9]] |
| Data sources/ measurement | 8* | | For each variable of interest, give sources of data and details of methods of assessment (measurement). Describe comparability of assessment methods if there is more than one group [[PAGE 6]] |
| Bias | 9 | | Describe any efforts to address potential sources of bias [[NA]] |
| Study size | 10 | | Explain how the study size was arrived at [[PAGE 7]] |
| Quantitative variables | 11 | | Explain how quantitative variables were handled in the analyses. If applicable, describe which groupings were chosen and why [[PAGE 7-9]] |
| Statistical methods | 12 | | (*a*) Describe all statistical methods, including those used to control for confounding |
|  |  |  | (*b*) Describe any methods used to examine subgroups and interactions |
|  |  |  | (*c*) Explain how missing data were addressed |
|  |  |  | (*d*) If applicable, explain how matching of cases and controls was addressed |
|  |  |  | (*e*) Describe any sensitivity analyses [[[PAGES 9-10]]] |
| **Results** | | | |
| Participants | 13* | | (a) Report numbers of individuals at each stage of study—eg numbers potentially eligible, examined for eligibility, confirmed eligible, included in the study, completing follow-up, and analysed [[PAGE 10]] |
|  |  |  | (b) Give reasons for non-participation at each stage [NA] |
|  |  |  | (c) Consider use of a flow diagram [[PAGE 11]] |
| Descriptive data | 14* | | (a) Give characteristics of study participants (eg demographic, clinical, social) and information on exposures and potential confounders [[PAGE 12]] |
|  |  |  | (b) Indicate number of participants with missing data for each variable of interest [[PAGE 11]] |
| Outcome data | 15* | | Report numbers in each exposure category, or summary measures of exposure [[PAGE 13, 15]] |
| Main results | 16 | | (*a*) Give unadjusted estimates and, if applicable, confounder-adjusted estimates and their precision (eg, 95% confidence interval). Make clear which confounders were adjusted for and why they were included [[PAGE 13-15]] |
|  |  |  | (*b*) Report category boundaries when continuous variables were categorized[[NA]] |
|  |  |  | (*c*) If relevant, consider translating estimates of relative risk into absolute risk for a meaningful time period [[NA]] |
| Other analyses | | 17 | Report other analyses done—eg analyses of subgroups and interactions, and sensitivity analyses [[PAGE 14-15]] |
| **Discussion** | | | |
| Key results | | 18 | Summarise key results with reference to study objectives [[PAGE 16]] |
| Limitations | | 19 | Discuss limitations of the study, taking into account sources of potential bias or imprecision. Discuss both direction and magnitude of any potential bias [[PAGE 18]] |
| Interpretation | | 20 | Give a cautious overall interpretation of results considering objectives, limitations, multiplicity of analyses, results from similar studies, and other relevant evidence [[PAGE 18]] |
| Generalisability | | 21 | Discuss the generalisability (external validity) of the study results [[PAGE 16-18]] |
| **Other information** | | | |
| Funding | | 22 | Give the source of funding and the role of the funders for the present study and, if applicable, for the original study on which the present article is based [[PAGE 21]] |

*Give information separately for cases and controls.

**Note:** An Explanation and Elaboration article discusses each checklist item and gives methodological background and published examples of transparent reporting. The STROBE checklist is best used in conjunction with this article (freely available on the Web sites of PLoS Medicine at http://www.plosmedicine.org/, Annals of Internal Medicine at http://www.annals.org/, and Epidemiology at http://www.epidem.com/). Information on the STROBE Initiative is available at http://www.strobe-statement.org.
